# Managing the Systemic Impact of Periodontitis

**DOI:** 10.3390/medicina58050621

**Published:** 2022-04-29

**Authors:** Giuseppe Mainas, Mark Ide, Manfredi Rizzo, Antonio Magan-Fernandez, Francisco Mesa, Luigi Nibali

**Affiliations:** 1 Periodontology Unit, Centre for Host-Microbiome Interactions, Dental Institute, King’s College London, London SE1 9RT, UK; giuseppe.mainas@kcl.ac.uk (G.M.); mark.ide@kcl.ac.uk (M.I.); 2 Department of Health Promotion, Mother and Child Care, Internal Medicine and Medical Specialties, School of Medicine, University of Palermo, 90133 Palermo, Italy; manfredi.rizzo@unipa.it; 3 Department of Periodontics, School of Dentistry, University of Granada, 18071 Granada, Spain; amaganf@ugr.es (A.M.-F.); fmesa@ugr.es (F.M.)

**Keywords:** periodontitis, inflammation, biomarkers, quality of life, public health, cardiovascular diseases, pregnancy outcome, diabetes mellitus, metabolic syndrome, psoriasis

## Abstract

Periodontitis is a microbially driven host-mediated disease that leads to loss of periodontal attachment and bone. It is associated with elevation of systemic inflammatory markers and with the presence of systemic co-morbidities. Furthermore, periodontal treatment leads to a 24–48 h-long acute local and systemic inflammatory response. This systemic response might increase the burden of patients with compromised medical history and/or uncontrolled systemic diseases. The correlation between periodontitis and systemic diseases, the impact of periodontitis on the quality of life and public health, the effects of periodontal treatment on systemic health and disease, and the available methods to manage systemic inflammation after periodontal therapy are discussed. The main focus then shifts to a description of the existing evidence regarding the impact of periodontitis and periodontal treatment on systemic health and to the identification of approaches aiming to reduce the effect of periodontitis on systemic inflammation.

## 1. Introduction

Periodontitis is a microbially driven host-mediated disease that leads to loss of periodontal attachment and bone [1] At first stage, gingival inflammation (gingivitis) is caused by bacterial biofilm formation. Consequently, progression of periodontal disease to destructive periodontitis depends on microbial dysbiosis in response to nutrients from gingival inflammatory and tissue breakdown products favouring the growth of some bacterial species, and also in response to anti-bacterial mechanisms that try to contain the microbial challenge within the gingival sulcus [1]. According to the 2017 World Workshop on the Classification of Periodontal and Peri-Implant Diseases and Conditions, severity and complexity of periodontitis are assessed following a staging process (Stage I, II, III and IV) that consider the initial clinical attachment level (CAL) whereas, the direct or indirect rate of progression is based on a grading scale (Grade A, B and C) that involves radiographic bone loss (RBL) and presence of risk factors such as smoking and diabetes [1].

However, periodontal inflammation is not just a local phenomenon. The possible link between periodontitis and systemic conditions has been extensively reported in 2013 in the Proceedings of a Workshop jointly held by the European Federation of Periodontology and the American Academy of Periodontology, where authors agreed that pro-inflammatory (infectious) events related to periodontitis may have a systemic impact and, vice-versa, some systemic disorders may influence periodontal outcomes [2,3,4].

Several studies have reported that both acute local and systemic inflammatory response is increased up to 24–48 h after periodontal treatment, either surgical or non-surgical [5,6]. Since this might be additional to the pre-existing inflammatory burden of patients with compromised medical history and/or uncontrolled systemic diseases, some have tried to investigate how to manage and modulate the impact of periodontal treatment on systemic inflammation.

Therefore, the present narrative review focuses on describing the existing evidence on (i) the impact of periodontitis and periodontal treatment on systemic health and (ii) effective measures to modulate the effect of periodontal treatment on systemic inflammation. Furthermore, the link between periodontitis and systemic diseases, the impact of periodontitis on the quality of life and public health and the effects of periodontal treatment on other co-morbidities will be covered in this article.

## 2. Periodontitis and Systemic Diseases

**Figure 1:**
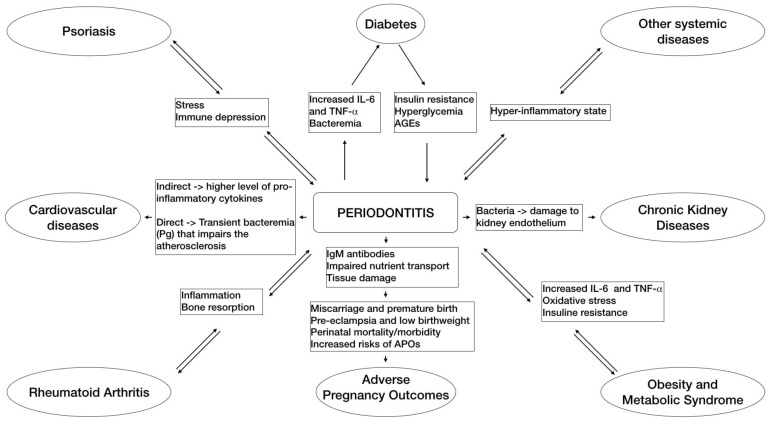
This flowchart schematically summarizes the main pathways of associations between periodontitis and some related systemic diseases based on the emerging evidence.

Describing in detail the complex mechanisms concerning the connection between periodontitis and systemic disease is not the aim of the present narrative review. For in-depth reading, we refer to the appropriate articles [7]. However, a brief introduction is provided in order to give the reader a general understanding of the subject (Figure 1). 

Two main simultaneous and not independent pathways, infection and inflammation, are involved in the pathogenic process of periodontitis–systemic connections [8]. The infectious pathway consists of oral micro-organisms that, together with putative pathogens located in periodontal pockets, may pass into the blood stream or respiratory tract causing a bacteremia that, in turn, can lead to complications for unhealthy and immunocompromised subjects, either systemically or possibly after selective colonization of distant sites and organs. On the other hand, the inflammatory pathway pertains to the systemic inflammation provoked by local and systemic effects of bacterial products and inflammatory molecules of periodontal origin that might be a risk factor for inflammatory associated systemic diseases in susceptible hosts [8]. 

As summarized in the Proceedings of a Workshop jointly held by the European Federation of Periodontology and the American Academy of Periodontology [2], periodontal disease has been associated with diabetes [3,9,10], cardiovascular diseases (CDV) [11,12,13,14,15], adverse pregnancy outcomes [4,16,17], obesity [18,19], metabolic syndrome [20,21], chronic obstructive airways disease [22,23], cognitive impairment such as Alzheimer’s [24,25], rheumatoid arthritis [26], chronic kidney diseases [27,28], psoriasis [29], depression [30], osteoporosis [31,32] and cancers [7,33]. The understanding of these associations is further complicated by the fact that several of these relationships may be bidirectional or even interrelated between systemic outcomes.

## 3. Impact of Periodontitis on Quality of Life and Public Health

Oral health is a functional, structural, aesthetic, physiological and psychological state of wellbeing and is essential to an individual’s general health and quality of life [34]. Almost two decades ago, at the 2003 World Workshop on Emerging Science in Periodontology, it was recognized that patient-based outcomes (PBO) had to be considered a research priority [35]. It has been reported that periodontal disease impairs oral health-related quality of life (OHRQoL) [36] and, in addition, a correlation between extent and severity of periodontitis exists [37]. Two systematic reviews found that non-surgical periodontal treatment (NSPT), now termed professional mechanical plaque removal (PMPR), moderately improved the OHRQoL of adult patients in the short-term (1 week) and long-term (12 months) specifically by reducing pain, psychological discomfort and physical disability [38,39], whereas a surgical approach had a relatively lower impact [38]. Another important aspect is correlated to the devasting impact that periodontal disease has on public health [40]. Jeffcoat et al. analyzed medical and dental insurance claims data from a large cohort who simultaneously experienced periodontitis and one systemic condition, including coronary artery disease, cerebrovascular disease, pregnancy, diabetes mellitus type 2 and rheumatoid arthritis. They concluded that, with the exception of those with rheumatoid arthritis, patients who were periodontally treated generated lower medical costs and fewer hospitalizations, with cost reductions of 72.7%, 40.9%, 40.2% and 10.7% for pregnancy, cerebrovascular disease, diabetes mellitus type 2 and coronary artery disease care, respectively [41].

In 2021, an Economist Intelligence Unit’s report (promoted by the European Federation of Periodontology/EFP) across six European countries that share similar conditions (France, Germany, Italy, the Netherlands, Spain and UK) was released [42]. Based on the concepts of the economic burden and return-on-investment (ROI), it highlighted that home care by patients aiming at preventing periodontal disease was very cost effective for society and ultimately reduced the need for more difficult and expensive treatment. It was inferred that this may also then indirectly significantly lower costs related to other systemic co-morbidities [42].

## 4. Effects of Periodontal Treatment on Systemic Health in Both the Short- and Long-Term

Risk factor control, oral hygiene instructions and supra- and sub-gingival tooth debridement (professional mechanical plaque removal or PMPR) form the hallmark of initial periodontal treatment, now codified as step 1 and 2 of periodontal treatment [43]. The possible mechanisms that link periodontal treatment and systemic inflammation could be that both the bacteremia and the tissue damage (trauma) after subgingival debridement determine an increase of pro-inflammatory mediators, such as Interleukin 1-beta (IL-1β) and 6 (IL-6), which ultimately induce liver production of acute-phase proteins [5,6,44].

A very interesting study described the kinetics of serum inflammatory markers following non-surgical (full-mouth) and surgical periodontal treatment in 14 patients with chronic periodontitis [5]. C-reactive protein (CRP), leucocyte counts, serum amyloid-A (SAA), D-dimers and cystatin C (renal function) were studied at 1, 7, 30, 90 and 180 days. A consistent increase in the serum level of CRP and SAA was observed at day 1 after PMPR and a smaller increase after the first surgery; D-dimer plasma levels significantly increased 24 h following PMPR but decreased after surgery, whereas cystatin C presented a moderate initial increase followed by an important reduction 12 months after treatment completion. Therefore, within the limitations of the study. such as small sample size and absence of control group, authors concluded that (i) both surgical and non-surgical periodontal treatment lead to systemic inflammation; however, (ii) healthy patients usually do not present any complications [5]. One of the most interesting aspect of these findings is that CRP serum levels were higher after PMPR compared to after surgical interventions, which might be counter-intuitive given the clinical perception of a greater trauma caused by surgery. A possible explanation might be the fact that the traumatized area is smaller in surgery than the full-mouth non-causal therapy, with a consequent reduced systemic inflammation due to a smaller post-operative wound. Another hypothesis is that subgingival debridement provokes a greater bacteremia, considering that the surgical treatment is provided after the same PMPR has reduced and modified the pathogens’ habitat, including number and quality of pathogens. Alternatively, a prerequisite for surgical intervention is the achievement of good oral hygiene, and this may also impact on local tissue inflammation, permeability and the degree of bacteremia. These short-term effects have been used as an experimental model to illustrate what might happen at a lower grade, more chronic and frequent exposure event every day in periodontitis patients, as well as an example to study the systemic acute-phase response [6].

Moreover, in the short- and long-term, other events have been detected [45,46]. A randomized clinical trial comparing the standard cycle of supragingival mechanical scaling and polishing and full-mouth intensive subgingival debridement observed that intensive treatment provoked an acute systemic inflammation (significantly higher levels of CRP, IL-6, soluble E-selectin and von Willebrand factor) and endothelial dysfunction (lower flow-mediated dilatation) at 24 h. Nevertheless, at 3- and 6 months, intensive treatment showed reduced indexes of periodontal disease severity and significantly better endothelial function [46]. These finding were corroborated by an observational study that found moderate acute systemic inflammation and endothelial dysfunction 1- and 7 days after providing an intensive periodontal treatment [45]. In addition, the same authors found an alteration of the hemostatic system in terms of increasing blood coagulability (significant increase of D-dimer levels) [45].

A very recent systematic review confirmed the aforementioned findings. The authors reported that conventional and, mainly, intensive PMPR provokes changes in CRP including a sharp increase over the first week [47].

The effects of periodontal treatment on people with systemic diseases, according to the best existing evidence, are described as follows.

### 4.1. Diabetes

Diabetes mellitus and periodontitis have a very complex bidirectional relationship that involves genetic factors and pro-inflammatory mediators [3]. Presence of periodontitis might impair insulin resistance which appears as hyperglycemia that produces advanced glycation end products (AGEs). In turn, AGEs lead to overproduction of IL-6, IL-1 and Tumor Necrosis Factor alpha (TNF-α). As a consequence, presence of AGEs in tissues causes impaired wound healing (improper collagen turnover in fibroblasts) and excessive neutrophil response to periodontal bacteria [48].

Considering the effect of inflammation in Diabetes mellitus type 2, it is thought that periodontal therapy may have a positive effect in reducing insulin resistance with following improvement of glycemic control [49]. Another study, with the aim of assessing the effects of PMPR on obese type 2 diabetes mellitus patients, evaluated inflammatory biomarkers such as CRP, IL-4, IL-6, IL-8, IL-10, TNF-α and fibrinogen. After 3 months, TNF-a and fibrinogen decreased significantly and, in general, the other biomarkers presented a trend towards reduction, even if not statistically significant [50]. A systematic review and meta-analysis found a statistically significant mean difference in favor of patients undergoing PMPR compared with the control group for CRP (−1.28 mg/L) and TNF-a (−1.33 pg/mL) after a follow-up period of from 1–12 months [51]. Another more recent systematic review including studies with follow-ups ranging between 3–6 months reported that PMPR contributes to the reduction of IL-6 serum levels in patients with type 2 diabetes mellitus [52].

### 4.2. Cardiovascular Diseases

Periodontal disease and atherosclerotic vascular disease (the main cause of cardiovascular disease) present an established association, as was stated in the 2009 Consensus by the editors of the American Journal of Cardiology and Journal of Periodontology, and in the 2012 Statement by the American Heart Association [53,54]. Several mechanisms have been proposed to explain this very close association, such as the infection of atherosclerotic plaques by periodontal pathogens, the pro-atherogenic effect on the lipid profile, and the systemic dissemination of pro-inflammatory mediators [55].

Two linking pathways have been extensively investigated: indirect and direct. The indirect pathway was described as the systemic inflammation provoked by periodontitis that leads to higher levels of several pro-inflammatory cytokines [56]. The direct pathway consists of periodontal pathogens that enter into the circulatory system and cause a transient bacteremia (e.g., toothbrushing, chewing, etc.) that can affect cardiovascular mediators and contribute to impair the atherosclerosis; Porphyromonas gingivalis (Pg), in particular, is capable of invading host cells such as macrophages, epithelial cells and fibroblasts [57].

Several systematic reviews have been carried out regarding the effect of periodontal treatment on cardiovascular disease markers [11,58,59]. One reported a significant reduction in almost each biomarker assessed in favor of treatment group (PMPR) compared to control group (no periodontal treatment) and, in addition, a sub-analysis related to patients with co-morbidities (diabetes and CVD) revealed that periodontal treatment reduced CVD risk factors [58]. Concerning CRP levels, a statistically mean difference of −0.231 mg/L, eight weeks after PMPR, was observed [59]. Other authors found that short-term periodontal treatment leads to local and systemic inflammatory response with disruption of the hemostatic system and endothelial function. After 6 months, a progressive decrease in CVD risk biomarkers including CRP, lipids, fibrinogen and E-selectin was observed [11].

Furthermore, two randomized clinical trials assessing the importance of an adequate and immediate PMPR on CVD surrogate markers have been published [46,60]. Compared to patients that only received supragingival mechanical scaling and polishing, patients who received an intense full-mouth subgingival plaque-removal treatment presented, at 24 h, a significant reduction in flow-mediated dilatation (the vasodilation of the brachial artery to assess the endothelial function), and a significant increase in the levels of CRP, IL-6, and the endothelial-activation markers, soluble E-selectin and von Willebrand factor. Six months later, however, intensive-treatment patients presented greater flow-mediated dilatation and lower E-selectin levels than the other group. The remaining parameters and biomarkers showed no statistical difference at 6 months. Therefore, the 1authors concluded that benefits in oral health are provided over time and lead to an improved endothelial function [46]. In the other study, periodontal patients with nonresponsive arterial hypertension were divided in two groups: the test group received immediate non-causal therapy, whereas the control group was treated 3 months later. It was found that the biomarkers assessed (CPR, IL-6 and fibrinogen) simultaneously and significantly decreased in plasma level, 3 months after the treatment [60]. Very interestingly, a prospective study on a Korean cardiovascular health population showed that presence of periodontal disease was associated with an increased risk of future stroke, acute myocardial infarction, hearth failure (major cardiovascular events) and death [61]. These findings were confirmed in a recent comprehensive systematic review and meta-analysis [62].

Other authors have focused on the potential pro-atherogenic alterations in plasma lipids and lipoproteins, since dyslipidemia is a common feature of patients with periodontitis; a systematic review and meta-analysis of published studies revealed that periodontitis is associated with higher plasma levels of total- and LDL-cholesterol and triglycerides, with a concomitant decrease of HDL-cholesterol concentrations [63]. Yet, beyond the increased concentrations of LDL-cholesterol, untreated periodontitis is also associated with an altered LDL subclass profile, with a predominance of atherogenic small dense LDL [64]; this means, therefore, that patients with periodontitis have alterations in both the quantity and the quality of LDL, which represents a well-known mechanism for the development of pro-atherogenic alterations and the enhancement of cardiovascular risk [65].

### 4.3. Adverse Pregnancy Outcomes

The association between periodontitis and adverse pregnancy outcomes (APOs) was reported to be not very strong due to conflicting results. A systematic review described the possible mechanisms that might justify this relationship [16]. In brief, (i) periodontal pathogens my pass into the placenta and disseminate to the fetal circulation and amniotic fluid, stimulating a fetal immune/inflammatory response that consists of production of IgM antibodies against the pathogens and the secretion of elevated levels of inflammatory mediators, that in turn might lead to miscarriage and premature birth. Furthermore, (ii) infection/inflammation may cause placenta structural changes provoking pre-eclampsia and impaired nutrient transport leading to low birthweight. In addition, (iii) fetal exposure may result in tissue damage, with the increased risk for perinatal mortality/morbidity. Besides, (iv) the elicited systemic inflammatory response might exacerbate local inflammatory responses at the feto-placental unit and increase the risk for APOs [16].

An RCT study on Indian patients evaluated CRP levels of pregnant women with periodontal disease. PMPR was provided during the second trimester of gestational period in the test group, whereas the control group did not receive any therapy. Those in the treatment group experienced statistically significant reductions in CRP values compared to control participants that had no significant reductions [66]. In another study, pregnant patients were randomly assigned to receive intensive PMPR either at 20–28 weeks of gestational age (GA) or postnatally. Treatment significantly reduced the gingival crevicular fluid (GCF) levels of IL-1, IL-6, IL-10, and IL-12p70 at 28 weeks GA compared with controls [67].

### 4.4. Obesity and Metabolic Syndrome

Obesity/overweight status and periodontal disease may have a bidirectional relationship as was reported by two systematic reviews [18,19]. In fact, both adipocytes and periodontal tissues, initiated by pathogens, can induce formation of pro-inflammatory cytokines (TNF-a, IL-6 etc.) that may exacerbate both conditions. In addition, periodontal tissues are thought to potentially worsen obesity through pathways involving the increased production of reactive oxygen species (ROS). Similarly, Metabolic Syndrome (MetS) and periodontitis have in common oxidative stress [21,32] and insulin resistance [68].

An intervention study on young Koreans evaluated the effects of a short-term weight control on inflammatory biomarkers in periodontally healthy subjects. It was found that caloric restriction might reduce IL-1b, Matrix Metalloproteinases 8 (MMP-8) and 9 (MMP-9) in GFC of obese patients [69]. Another study compared PMPR in patients with MetS and patients that were systemically healthy. Results showed that periodontal treatment decreased oxidative stress and circulating levels of hs-CRP and IL-6 in MetS patients but not in healthy subjects [70].

In a previous systematic review and meta-analysis, a close association was shown between MetS and periodontitis, and the authors therefore recommended that oral health assessments and periodontal diagnosis (evaluating probing pocket depths, periodontal attachment level, and bleeding on probing charts) should be part of the routine diagnostic procedures in MetS patients [71]. However, this still needs to be fully implemented in daily clinical practice.

### 4.5. Rheumatoid Arthritis

Even though periodontitis and rheumatoid arthritis share some features including inflammation and bone resorption, their relationship cannot be considered strongly evidence-based as yet [72]. Systematic reviews have found that erythrocyte sedimentation rate and CRP might be elevated in rheumatoid arthritis patients with periodontitis; besides, presence of serum antibodies for periodontal pathogenic bacteria such as Porphyromonas gingivalis (Pg) has been detected in the synovium [26,72]. The presence of Pg may also lead to rheumatoid arthritis due to its role in triggering the production of anticitrullinated protein antibodies [73].

A systematic review showed a reduction in erythrocyte sedimentation rate (mean difference of −0.479) and a trend towards reduction of TNF-a (−1.352, not statistically significant) after PMPR. Moreover, no significant differences were observed in terms of IL-6, anticitrullinated protein antibodies and rheumatoid factor [74].

### 4.6. Chronic Kidney Disease

Periodontitis and chronic kidney disease (CKD) present an established relationship due to the systemic inflammatory status and presence of bacteria in the bloodstream that may trigger damage to kidney endothelium and lead to CKD [27]. A systematic review and meta-analysis reported that dialysis patients who underwent PMPR presented significant decreased hs-CRP levels compared to untreated patients, whereas no significant difference was found related to IL-6 and albumin levels [75]. A study found that periodontal therapy led to significant reductions in CRP, IL-6 and pro-hepcidin in patients with chronic kidney disease at 3 months [76].

### 4.7. Psoriasis

Psoriasis and periodontal disease may be linked since they have in common an exaggerated immune response in epithelial surfaces with a dysregulation of the host inflammatory response [77], and have several risks factors such as stress and immune depression [78].

In a randomized clinical trial, subjects with psoriasis were randomly allocated into immediate (test) and delayed (control) PMPR. Two months after treatment, IL-2, IL-6 and secretory Immunoglobulin A (sIgA) levels dropped significantly only in the test group [79].

### 4.8. Other Systemic Diseases

Although potential associations between periodontitis and both cognitive decline and respiratory diseases have been reported [22,24], we are not aware of studies assessing change in inflammatory markers post-treatment in these specific conditions.

Overall, it has to be remarked that periodontal treatment reduces local and systemic inflammation in the long-term (at 6–12 months) as well as decreasing the risk of progression of the disease [47,80]. Consequently, one can argue that its benefits overcome the questionable adverse events in all periodontal patients, including the short-term acute-phase response post-treatment, independently of their systemic condition [5,81].

Although it may be advisable to try and reduce the short-term hyper-inflammatory effect post-periodontal treatment using new/experimental treatments, one might question this, as reducing this short-term inflammatory effect could be detrimental for the patient (in particular for systemically compromised subjects), as the early systemic inflammatory response may be a necessary response to injury. Unfortunately, besides those studies already discussed, no others investigating the possible side effects of periodontal therapy on compromised and/or systemic diseased patients are available in the existing scientific literature. Therefore, in the future, it is hoped that new studies will clarify this interesting and non-negligible aspect.

## 5. How We Can Reduce the Effect of Periodontal Treatment on Systemic Inflammation?

Given these short-term effects of periodontal treatment on systemic inflammation and associated claims of potentially increased cardiovascular events [82], it is legitimate to think about ways to treat periodontitis, while not incurring this risk. However, not many studies have investigated methods to manage systemic inflammation after periodontal therapy.

Table 1 summarizes studies investigating methods for modulation of systemic response to periodontal treatment. An investigation tried to modulate the inflammatory reaction after periodontal treatment [81]. Inflammatory cytokine production 24 h after treatment was compared in subjects undergoing full-mouth treatment versus quadrant scaling, and it was found that the subjects undergoing conventional quadrant scaling did not show a significant increase of CRP, IL-6 or TNF-α as compared to the ones undergoing full-mouth approach. On the other hand, in patients that underwent the full-mouth scaling, a strong systemic inflammatory reaction was observed. Furthermore, it was observed that one of the factors that was associated with the systemic response was the overall treatment time. Thus, it was suggested that, in subjects with complex medical histories and/or uncontrolled comorbidities, a conventional treatment approach and shorter appointments should be preferred in order not to cause an intense systemic inflammatory reaction [81]. Nevertheless, it is very important to remark that some other patients should be ideally treated in the minor number of appointments, since they might need antibiotics prophylaxis (e.g., endocarditis) [83] or other preparation prior to the treatment (e.g., hematological disorders and cardiovascular diseases) [84,85,86].

A randomized placebo-controlled clinical trial tested the possible adjunct effect of folic acid (FA) provided together with PMPR on homocysteine (Hcy) and CRP levels in GCF of periodontal patients. Although the FA group showed significant clinical attachment level gain, there was no significant change in biochemical parameters compared to placebo [87]. In support of these findings, low level serum and insufficient dietary FA intake showed impairment of periodontal health, in particular in smoker patients [92], whereas FA supplements and mouth-rinses led to a significant reduction of gingival inflammation and overgrowth [93].

Another study evaluated the effect of long-term weekly supragingival irrigations with aerosolized 0.5% hydrogen peroxide as maintenance therapy followed by PMPR on hs-CRP and white blood cell (WBC) count assessed at baseline and at 1, 2 and 3 years. After 1- and 2 years follow-up, the plasma levels of inflammatory markers decreased significantly, whereas at 3 years a less evident decrease was found, with values almost similar to the ones recorded after 2 years [88]. In light of the results, supragingival irrigations with 0.5% hydrogen peroxide may be considered a promising method. In fact, as has been previously reported, supragingival irrigations can reduce the number of periodontal pathogens [94], bacterial toxins [95] and host inflammatory products derived from periodontitis [23]. In contrast, a split-mouth study comparing 0.02% chlorhexidine (CHX) irrigations with sterile water, showed that CHX irrigations did not have any influence on increasing levels of lipopolysaccharide (LPS) and IL-6, 5 min and 120 min after ultrasonic instrumentation, respectively [89].

Concerning the important of maintenance, a study observed that, despite an immediate reduction of systemic inflammation, lack of maintenance results in a long-term return to the baseline levels of inflammation [96].

Rasperini and et al. in 2019 assessed the effect of micronutrient supplements on systemic and local inflammatory markers, including CRP and salivary MMPs-8/-9. All patients followed the Mediterranean Diet (MD), characterized by the high consumption of olive oil, vegetables and fruits rich in antioxidants and vitamins; together with PMPR, one group received a micronutrient complex and the other olive oil-filled capsules. After one month, the patients underwent a full-mouth session of PMPR. No statistically significant differences were observed between groups in terms of salivary and serum MMP-8/-9 levels at any time point (baseline, 1- and 3 months post-treatment). Concerning the intra-group analysis, in the olive oil group the reduction of MMP-8/-9 in saliva was not significant at 1 and 3 months; in the other group, MMP-8/-9 levels were significantly reduced at 3 months, whereas at 1 month only MMP-9 levels decreased significantly. CRP serum levels were reduced after 3 months in both groups, but not enough to reach statistical significance. Overall, even though micronutrients are suspected to provide a beneficial effect on periodontal tissues by reducing inflammation and damage due to inflammatory diseases [97], MD did not affect local and systemic inflammatory parameters, except for a slight decrease in MMP saliva levels [90].

Graziani et al. in 2019 compared PMPR with application of enamel matrix derivative (EMD) (test group) versus PMPR (control group) in order to compare acute phase (at 24 h) and medium-term (3 months) inflammation in periodontal patients. Serum glucose, lipids, hs-CRP, cystatin C, D-dimer and fibrinogen were studied. Both treatments resulted in a marked increase in systemic inflammation at 24 h, with significant increase of CRP, D-dimer and cystatin C in the control group and only an increase in CRP and fibrinogen in the test group. D-dimer only showed a significantly higher increase in control group 24 h after treatment. At 3 months, biomarker levels significantly dropped and returned to baseline values in both groups, except for glucose, which was significant lower in the test group. The authors concluded that the adjunctive use of EMD led to a lower fibrinolysis. This may be due to the anti-inflammatory effect that EMD has, acting as a blood clot stabilizer and, consequently, improving wound healing [91].

## 6. Conclusions and Recommendation for Future Research

Within the limitations of the studies included in the present review and considering the existing paucity in the literature, some conclusions can be drawn.

Periodontitis has an impact on systemic inflammation.

Periodontal treatment has an impact on systemic inflammation, both short-term (acute-phase response with increase in inflammation) and long-term (possible reduction in systemic inflammation). This may vary according to existing co-morbidities.

Modulation (reduction) of the acute-phase response might be achieved by dividing periodontal treatment into more sessions, thus reducing treatment time for each session. However, when an antibiotic prophylaxis (endocarditis) and other special prior preparations (bleeding and cardiovascular disorders) are required, considerations should be given regarding the total number of appointments that might be reduced. Therefore, after carefully assessing their medical history, patients have to receive a tailored treatment plan.

Modulation (increased reduction) of the long-term benefits on inflammation might be achieved by performing the periodontal treatment itself and, very importantly, by providing adequate periodontal maintenance (e.g., regular recall intervals).

Overall, there is need for studies with long-term follow-ups, larger sample size and robust methodology to reinforce the scientific evidence of the management of inflammation after periodontal treatment.

## Figures and Tables

**Table 1:** Brief summary of studies investigating methods to reduce the systemic impact of professional mechanical plaque removal (PMPR).

Authors	Study	Periodontitis Definition	Follow-up	Treatment(s)	Results
Graziani et al., 2015 [81]	Randomized clinical trial	Proximal attachment loss of ≥3 mm in ≥2 non-adjacent teeth (Tonetti & Claffey 2005), bleeding on probing on at least 25% of their total sites, and documented radiographic bone loss	3 months	Full-mouth vs. quadrant scaling	Full-mouth PMPR resulted in significant increase of CRP, IL-6 or TNF-α compared to quadrant (conventional) PMPR
Keceli et al., 2020 [87]	Randomized placebo-controlled clinical trial	Periodontitis stage II–III (2017 World Workshop)	6 months	PMPR + Folic acid (FA) + vs. PMPR alone	FA group resulted in no significant change in homocysteine (Hcy) and CRP levels in GCF compared to placebo group
Zekonis et al., 2016 [88]	Prospective cohort study	PPD ≥ 6 mm on at least 2 teeth, and radiographic evidence of horizontal and vertical bone loss.	1-, 2- and 3-years	PMPR + supragingival irrigations with 0.5% hydrogen peroxide	Plasma levels of high sensitivity CRP (hs-CRP) and white blood cell (WBC) count decreased significantly after 1 and 2 years, whereas at 3 years a less evident decrease was found
Lee et al., 2008 [89]	Prospective single-masked, split-mouth, crossover interventional study	At least five sites per quadrant with PPD ≥ 5 mm and radiographic evidence of alveolar bone loss.	Day 0	PMPR (ultrasonic instrumentation) + 0.02% chlorhexidine (CHX) irrigations with sterile water	CHX irrigations did not have any influence on increasing levels of lipopolysaccharide (LPS) and IL-6
Rasperini et al., 2019 [90]	Randomized clinical trial	at least two sites with PPD > 7 mm, bleeding on probing > 25%	Day 0, 1 month and 3 months	PMPR + macronutrient complex vs. PMPR + olive oil-filled capsules	No statistically significant differences were observed between groups in terms of salivary and serum MMP-8/-9 levels at any time point. CRP serum levels were reduced after 3 months in both groups, but not significantly
Graziani et al., 2019 [91]	Randomized clinical trial	AL of ≥3 mm in ≥2 non-adjacent teeth (Tonetti, Claffey, & European Workshop in Periodontology Group C, 2005), bleeding on probing on at least 25% of total sites and documented radiographic bone loss	3 months	PMPR + EMD vs. PMPR	At 24 h significant increase of CRP, D-dimer and cystatin C in control group and, only increase in CRP and fibrinogen in EMD group. At 3 months, biomarkers levels significantly decreased and returned to baseline values in both groups, except for glucose that was significant lower in the EMD group

PPD = probing pocket depth; AL = attachment loss; PMPR: professional mechanical plaque removal; CHX: chlorhexidine; EMD: Emdogain.
